# Comorbidities, clinical outcome and rate of herpes simplex positive PCR in patients with keratitis, corneal erosions and ulcers

**DOI:** 10.1186/s12348-025-00515-4

**Published:** 2025-08-07

**Authors:** Mhd Hosam Dandachli, Anna-Karina B. Maier, Jörg Hofmann, Tina Dietrich-Ntoukas

**Affiliations:** 1 Department of Ophthalmology, Charité – Universitätsmedizin Berlin, Corporate Member of Freie Universität Berlin and Humboldt-Universität zu Berlin, Augustenburger Platz 1, Berlin, 13353 Germany; 2 Department of Ophthalmology, University Hospital Jena, Friedrich Schiller University Jena, Am Klinikum 1, Jena, 07747 Germany; 3 Institute of Virology, Charité – Universitätsmedizin Berlin, Corporate Member of Freie Universität Berlin and Humboldt-Universität zu Berlin, Sylterstraße 2, Berlin, 13353 Germany

**Keywords:** Herpes simplex keratitis, Persistent epithelial defects, Polymerase chain reaction, Comorbidities, Complications, Eye and corneal surgery, Clinical outcome, Diabetes mellitus

## Abstract

**Introduction:**

Herpes simplex keratitis (HSK) is a recurrent infection with a high risk of corneal blindness. The aim of the study is to investigate the HSV-PCR-positive smear rate, the ocular and systemic comorbidities and the impact of these comorbidities on the clinical outcome in a group of patients with pathologic corneal findings such as keratitis, persistent epithelial defects and corneal ulcers.

**Methods:**

In this retrospective study, we recruited 194 eyes who underwent PCR testing for HSV-1 DNA in our tertiary eye clinic from 2015 to 2021 due to suspected HSK. A poor outcome was defined as final visual acuity > 0.4 according to the Logarithm of the Minimum Angle of Resolution (LogMAR) or the need for at least one corneal surgery.

**Results:**

HSV-1-DNA was detected in 18.6% of the eyes. Corneal scarring (28.8%) and persistent epithelial defects (PED) (8.5%) were the most common complications. The highest recurrence rate (11.6%) was documented 3 months after sampling. 41.2% received systemic antiviral therapy at the first visit after collecting the sample. 75 eyes (38.7%) required at least one corneal surgery, of which amniotic membrane transplantation was the most common corneal procedure in 45 eyes (23.2%). 151 eyes (77.8%) had at least one ocular comorbidity, including previous ocular and corneal procedures (32% and 19.1% respectively) and blepharitis (26.3%). The most common systemic comorbidities were atopic diseases (10.8%), systemic immunosuppression (9.3%) and diabetes mellitus (8.8%). Previous ocular and corneal surgery, glaucoma and diabetes mellitus correlated with a poor outcome (*P* < 0,001). The average BCVA at the last follow-up (0.76 ± 0.83 LogMAR) was significantly better than at the time of sample collection (0.94 ± 0.76 LogMAR) (*P* < 0.001).

**Conclusion:**

Our data confirm that HSK should be treated based on clinical findings regardless of the PCR result. We demonstrate for the first time, that comorbidities are very common and especially previous ocular and corneal surgery, glaucoma and diabetes mellitus are associated with a poor outcome. Although corneal surgery was necessary in almost 40% of the eyes during the follow-up due to a complicated course, a significant overall improvement in visual acuity was achieved compared to the initial findings.

## Introduction

Herpes simplex keratitis (HSK) is a recurrent infection with a high risk of corneal blindness. Its incidence per 100,000 persons per year varies between countries and amounts to 31.5 in France and 11.8 in Minnesota, USA [1, 2]. The treatment costs of HSK in the USA in the year 2003 were estimated to have reached 17.7 million US dollars, indicating the huge financial burden of the disease [3]. The diagnosis of HSK can be challenging because clinical manifestations do not always match the typical HSK-findings. Many diagnostic tools have been used to confirm the diagnosis, with polymerase chain reaction (PCR) being the gold standard. Nevertheless, PCR does not provide absolute diagnostic certainty and therefore treatment should be based on clinical diagnosis.

Persistent epithelial defects (PED) refer to slow corneal wound healing that lasts longer than 10–14 days despite treatment [4, 5]. This wound healing disorder can be caused by various factors, including basement membrane dystrophies [4], limbal stem cell insufficiency [6], infectious keratitis, corneal nerve trauma after ocular surgery or autoimmune diseases [4]. In addition, HSK complications such as corneal ulcers, metaherpetic keratitis and neurotrophic keratopathy (NK) can contribute to the development of PED [7]. NK is a degenerative disease characterized by reduced or absent corneal sensitivity, which can cause PED. Many ocular causes of NK have been described including recurrent HSK, toxic eye drops, physical and chemical burns, the use of contact lenses and previous ocular surgeries [8]. Multiple comorbidities have been reported in the literature to be risk factors for HSK and PED. These include ocular trauma, ocular surface disease, corneal and ocular surgery, atopic diseases, diabetes mellitus, and immunosuppression [9–15].

Differential diagnoses of HSK include other viral keratitis, including herpes zoster keratitis and keratitis epidemica, microbial keratitis caused by bacteria, acanthamoeba or fungi, contact lens-associated keratitis, toxic epitheliopathy and tyrosinemia-associated keratitis [16]. This last entity is difficult to distinguish from the HSK due to its pseudodendritic appearance [17].

The aim of the study is to investigate the HSV-PCR-positive smear rate, ocular and systemic comorbidities and the possible influence of these comorbidities on the clinical outcome in a group of patients with pathologic corneal findings such as keratitis, persistent epithelial defects and corneal ulcers.

## Methods

In this retrospective study, a total of 268 eyes were recruited from 259 patients who underwent PCR testing for HSV-1 DNA in our Department of Ophthalmology at the Charité - University Medicine as tertiary care eye clinic from 2015 to 2021 with corneal pathologies as keratitis, persistent epithelial defects and ulcers. In patients with bilateral involvement, the eye with minor corneal involvement was excluded. Collected materials included corneal smears, scrapings, conjunctival and lid smears and anterior chamber and vitreous punctures. Conventional qualitative PCR was performed in a standardized manner in the Institute of Virology at Charité– University Medicine Berlin. In addition, a further 65 eyes were excluded in which no corneal disease was present but involvement of other parts of the eye. Finally, 194 eyes were included. The corneal specialists of the department suspected putative HSK based on clinical presentation and indicated sample collection to confirm the diagnosis. However, many cases presented with unclear or complex pathologic corneal findings, in which HSK was not the main differential diagnosis. In general, diagnostics in cases of unclear infectious keratitis in our department include microbiological testing for different germs (culture, PCR) and additionally, confocal microscopy if indicated, especially in cases of putative acanthamoeba or fungal keratitis. The clinical examination was performed at baseline and at each follow-up visit and included the following parameters: best corrected visual acuity (BCVA) (measured after Snellen and converted to LogMAR), ocular pressure measurement, corneal sensitivity testing, anterior segment slit lamp examination, and fundoscopy. The therapy was applied according to the suspected clinical diagnosis and smear results. The study was conducted in accordance with the standards of the Declaration of Helsinki after approval was granted by the local ethics committee (EA2/071/24) of Charité - University Medicine Berlin. The LogMAR equivalent of BCVA measured after Snellen is presented in Table 1.

**Table 1:** LogMAR equivalent of BCVA measured after Snellen

Snellen decimal	LogMAR
2.00	−0.3
1.60	−0.2
1.25	−0.1
1.00	0
0.80	0.1
0.63	0.2
0.50	0.3
0.40	0.4
0.32	0.5
0.25	0.6
0.20	0.7
0.16	0.8
0.125	0.9
0.10	1
0.08	1.1
0.06	1.2
0.05	1.3
0.04	1.4
0.03	1.5
0.025	1.6
0.01/Counting fingers	1.9
Hand motion	2.3
Light perception	2.7
No light perception	3

The following variables were evaluated: the results of sample collecting, ocular and systemic comorbidities, clinical manifestations, applied therapy, recurrence form as well as complications at follow-up examinations and clinical outcome regarding best corrected visual acuity (BCVA) and the need for corneal surgery. A poor outcome was defined as final visual acuity > 0.4 according to Logarithm of the Minimum Angle of Resolution (LogMAR) (< 0.4 according to Snellen) or the need for at least one corneal surgery.

Concerning the statistical analysis, the following descriptive statistics were applied: Mean, median, standard deviation, percentages and range. The independent T-test was used to compare the continuous data. The chi-square test was used to compare categorial data. All data were analyzed using SPSS Statistics Version 29.0 (IBM Corp, Armonk, New York, USA). A *P*-value < 0.05 was considered significant.

## Results

### Baseline characteristics

The demographic data of the patients are summarized in Table 2. The mean age of the patients was 59.39 ± 22.3 (range 1 to 99), and the mean BCVA (logMAR) at onset was 0.938 ± 0.758. There was no significant difference in terms of gender distribution. Intraocular pressure (divided into 3 groups: low [0–9 mmHg], normal [10–21 mmHg] and high [> 21 mmHg]) was measured in 156 eyes. 3.8% of the eyes had an elevated intraocular pressure and 1.9% had a low intraocular pressure. The median follow-up time was 30 weeks (range 1 to 342). 177 patients visited the clinic at least once after sample collection.

**Table 2:** Demographic data of the patients at onset

Age	Mean ± SD	59.39 ± 22.261
	Range	1 bis 99
	Median	62
Gender	Male (%)	92 (47.4)
	Female (%)	102 (52.6)
Mean of BCVA (logMAR) ± SD		(*N* = 188) 0.938 ± 0.758
IOP	High (%)	(*N* = 156) 6 (3.8)
	Normal (%)	(*N* = 156) 147 (94.2)
	Low (%)	(*N* = 156) 3 (1.9)
Follow-up in weeks	Range	1 to 342
	Median	30

*BCVA* best corrected visual acuity, *logMAR* Logarithm of the Minimum Angle of Resolution, *SD* Standard deviation, *IOP* intraocular pressure

### Sampling results

Collected materials included corneal smears (83.5%), corneal scrapings (8.2%) and anterior chamber punctures (8.2%). HSV-1 DNA was detected in 36 eyes (18.6%). Among the remaining eyes, 147 eyes (75.8%) had a negative result, and 11 eyes (5.7%) remained without a result (due to failure of internal control, the negative HSV-1 PCR result was not reliable). In 18.6% of samples, other pathogens were detected: These included acanthamoeba, bacteria, fungi, skin flora and other viruses (rubella virus and varicella zoster virus). Bacteria were detected in 17 eyes (8.8%); four of these eyes also had a positive PCR result for HSV-1. The PCR results are shown in Tables 3 and 4.

**Table 3:** HSV-1 PCR results of the collected samples

PCR result	Number of patients (%)
Positive	36 (18.6)
Negative	147 (75.8)
Without a result	11 (5.7)
Bacteria	17 (8.8)
Skin flora	8 (4.1)
Fungi	7 (3.6)
Acanthamoeba	5 (2.6)
Other viruses	4 (2.1)

**Table 4:** Visualization of the detected pathogens depending on the HSV-1 PCR result

Other pathogens	HSV-1-result		
	Positive	Negative	Ø
Bacteria	4	13	0
Skin flora	0	7	1
Fungi	2	5	0
Acanthamoeba	0	5	0
Other viruses	1	1	2

Ø: HSV-1-PCR without a result

### Ocular comorbidities

The most common ocular comorbidity was non-corneal eye surgery, which included cataract, glaucoma, retinal and strabismus surgery. This was documented in 62 eyes (32%). The second most common comorbidity was blepharitis in 51 eyes (26.3%). Another common ocular comorbidity was corneal surgery in 37 affected eyes (19.1%). An unspecified previous eye infection was a risk factor, as 17% of patients reported this comorbidity at the time of sample collection. 10.8% of the eyes had a previously diagnosed glaucoma disease. Considering the total number of comorbidities per eye, 151 eyes (77.8%) had at least one and 75 eyes (38.7%) had at least two ocular comorbidities. An overview of the ocular comorbidities is shown in Table 5.

**Table 5:** Ocular comorbidities

Ocular comorbidities	Number of patients (%)
Non-corneal eye surgery	62 (32)
Blepharitis	51 (26.3)
Corneal surgery	37 (19.1)
Unspecific pervious eye infection	33 (17.0)
Glaucoma	21 (10.8)
Wear of contact lenses	19 (9.8)
Ocular Trauma	13 (6.7)
Dry eye disease	7 (3.6)
Other Keratopathy	7 (3.6)
Fuchs’ endothelial dystrophy	7 (3.6)
Unspecific conjunctivitis	4 (2.1)
Ocular Pemphigoid	3 (1.5)
Graft-versus-host disease	3 (1.5)
Limbal stem cell deficiency	3 (1,5)
Sjögren’s syndrome	2 (1.0)
Entropion	2 (1.0)
Thyroid Eye Disease	2 (1.0)
Keratoconus	2 (1.0)
Ectropion	1 (0.5)
Stenosis of the lacrimal duct	1 (0.5)
At least 1 Comorbidity	151 (77.8)
At least 2 Comorbidities	75 (38.7)

### Systemic comorbidities

79 patients (40.7%) had at least one and 22 patients (11.3%) had at least two systemic comorbidities. Atopic diseases, including bronchial asthma and neurodermatitis, were the most common systemic comorbidity with a frequency of 10.8%. The second most common comorbidity was systemic immunosuppression as reported in 9.3% of the patients. Diabetes mellitus was the third most common systemic comorbidity, accounting for 8.8% of patients. Rheumatic diseases came in fourth place with 11 affected patients (5.7%). 4.6% of patients had a previous tumor disease. An overview of systemic comorbidities is shown in Table 6.

**Table 6:** Systemic comorbidities

Systemic comorbidities	Number of patients (%)
Atopic disease	21 (10.8)
Immunosuppression	18 (9.3)
Diabetes mellitus	17 (8.8)
Rheumatic diseases	11 (5.7)
Previous tumor/oncologic disease	9 (4.6)
Rosacea	7 (3.6)
Psoriasis	5 (2.6)
Stem cell or organ transplantation	3 (1.5)
Previous extraocular Herpes infection	3 (1.5)
HLA-B27 positivity	3 (1.5)
Pityriasis rubra	1 (0.5)
Human immunodeficiency virus positivity	1 (0.5)
Acne	1 (0.5)
Methicillin resistant staphylococcus aureus	1 (0.5)
Chronic inflammatory bowel disease	1 (0.5)
Sarcoidosis	1 (0.5)
At least 1 Comorbidity	79 (40.7)
At least 2 Comorbidities	22 (11.3)

*HLA* human leukocyte antigen

### Clinical manifestations at onset

At the time of sample collection, corneal sensitivity was measured in 59 eyes and was reduced in 38 eyes (64.4%). Corneal erosion was documented in 131 eyes (67.5%), keratitis superficialis punctata in 46 eyes (23.7%), corneal infiltrate in 42 eyes (21.6%), corneal opacity in 55 eyes (28.4%), stromal edema in 38 eyes (19.6%), endothelial precipitates in 32 eyes (16.5%), corneal vascularization in 71 eyes (36.6%), and conjunctival injection in 121 eyes (62.4%). Further details on the clinical manifestations are shown in Table 7.

**Table 7:** Clinical manifestations at onset

Clinical manifestations	Number of patients (%)
Corneal erosion	131 (67.5)
Reduced corneal sensitivity	(*N* = 59) 38 (64.4)
Conjunctival injection	121 (62.4)
Corneal vascularization	71 (36.6)
Opacity	55 (28.4)
Superficial punctate keratopathy	46 (23.7)
Infiltrate	42 (21.6)
Stromal edema	38 (19.6)
Endothelial precipitates	32 (16.5)
Epithelial edema	7 (3.6)
Corneal thinning	6 (3.1)
Epithelial closure ridge	4 (2.1)
Epithelial bullae	3 (1.5)

### Complications at onset

On the day of sampling, corneal ulceration was documented in 38 eyes (19.6%), persistent corneal erosion in 28 eyes (14.4%), pannus formation in 5 eyes (2.6%) and scarring in 19 eyes (9.8%). An overview of all complications at onset is shown in Table 8.

**Table 8:** Complications at onset

Complications	Number of patients (%)
Corneal ulcer	38 (19.6)
Persistent corneal erosion	28 (14.4)
Scar formation	19 (9.8)
Elevated intraocular pressure	5 (2.6)
Pannus	5 (2.6)
Calcification	2 (1.0)
Corneal perforation/Descemetocele	2 (1.0)

### Topical and systemic therapy

The therapy was recorded at the first clinic visit after the sample collection. As mentioned above, only 177 patients had at least one follow-up. Acyclovir ointment was used in 31 eyes (17.5%), acyclovir tablets in 56 patients (31.6%), topical steroids in 82 eyes (46.3%), topical pressure-reducing drops in 27 eyes (15.3%), systemic steroid therapy in 11 patients (6.2%), methotrexate in 7 patients (4.0%), antibiotic eye drops in 139 eyes (78.5%), antifungal eye drops in 12 eyes (6.8%) and mydriatics in 20 eyes (11.3%). An overview of the therapy is shown in Table 9.

**Table 9:** Topical and systemic therapy at the first clinic visit after sample collection

**Therapy**		**Number of patients (%)** *N*=177	
Topical antiviral therapy	Ganciclovir	43 (24.3)	
	Acyclovir	31 (17.5)	
	Valaciclovir	2 (1.1)	
Systemic antiviral therapy	Acyclovir	56 (31.6)	
	Valaciclovir	17 (9.6)	
	Ganciclovir	0 (0)	
Topical steroids	Unpreserved	66 (37.3)	
	Preserved	16 (9.0)	
Systemic steroids		12 (6.8)	
Lubricant eye drops		151 (85.3)	
Anti-glaucomatous drops	Unpreserved	15 (8.5)	
	Preserved	12 (6.8)	
Systemic acetazolamide		5 (2.8)	
Ciclosporin drops		7 (4.0)	
Serum drops		1 (0.6)	
Systemic Immunosuppression	Prednisolone	11 (6.2)	
	MTX	7 (4.0)	
	TNF-α-Inhibitor	2 (1.1)	
	MMF	1 (0.6)	
	Tocilizumab	1 (0.6)	
	Sirolimus	1 (0.6)	
	Hydroxychloroquine	1 (0.6)	
Other topical therapy	Antibiotics	139 (78.5)	
	Mydriatics	20 (11.3)	
	Antimycotics	12 (6.8)	
	Antiseptic drops	9 (5.1)	
	Propamidin-Isethionat	8 (4.5)	
	Hyaluronic acid	4 (2.3)	

*MTX* Methotrexate, *MMF* Mycophenolate-Mofetil, *TNF* tumor necrosis factor

### Follow-up data

The new occurrence of infiltrates, epithelial bullae, corneal edema, endothelial precipitate and corneal vascularization was considered as HSK-recurrence. The highest recurrence rate (11.6%) was documented 3 months after sample collection. The scar formation had an average frequency of 28.8% and was the most common complication throughout the follow-up. The best BCVA was 0.870 ± 0.801 and was recorded 3 months after collecting the samples. Further details on the follow-up data are shown in Table 10.

**Table 10:** BCVA, recurrence rate and complications at follow-up examinations

**Time (number of patients)**		**3 months** **(129)**	**6 months** **(91)**	**12 months** **(70)**	**24 months** **(43)**	**36 months** **(18)**
**Data**						
Mean BCVA ± SD (logMAR)		0.870 ± 0.801	1.078 ± 0.850	1.046 ± 0.885	0.947 ± 0.851	0.929 ± 0.887
HSK-Recurrence (%)		15 (11.6)	4 (4.4)	4 (5.7)	2 (4.7)	1 (5.6)
Complications (%)	Ulcer	4 (3.1)	1 (1.1)	0 (0)	0 (0)	0 (0)
	Perforation/ Descemetocele	0 (0)	0 (0)	0 (0)	1 (2.3)	0 (0)
	Persistent erosion	24 (18.6)	13 (14.3)	5 (7.1)	1 (2.3)	0 (0)
	Elevated intraocular pressure	1 (0.8)	1 (1.1)	3 (4.3)	0 (0)	0 (0)
	Pannus	4 (3.1)	4 (4.4)	3 (4.3)	2 (4.7)	2 (11.1)
	Calcification	3 (2.3)	3 (3.3)	3 (4.3)	3 (7.0)	0 (0)
	Loose or spiky threads	3 (2.3)	2 (2.2)	0 (0)	0 (0)	0 (0)
	Graft rejection	1 (0.8)	1 (1.1)	0 (0)	0 (0)	0 (0)
	Step formation at the graft-host interface	0 (0)	1 (1.1)	1 (1.4)	0 (0)	0 (0)
	Flap dehiscence	0 (0)	0 (0)	0 (0)	0 (0)	0 (0)
	Scar formation	37 (28.7)	24 (26.4)	21 (30.0)	11 (25.6)	6 (33.3)

*BCVA* best corrected visual acuity, *SD* Standard deviation, *logMAR* Logarithm of the Minimum Angle of Resolution, *IOP* intraocular pressure

### Clinical outcome

The BCVA (logMAR) was measured in 185 eyes at the last clinic visit and showed a mean value of 0.775 ± 0.825. This differed significantly (*P*-value = < 0.001) from the average BCVA at the time of sampling. To investigate whether antiviral therapy improved the clinical outcome, we compared the BCVA in patients with clinical suspicion of HSK but negative HSV-PCR test (*n* = 60) before and after antiviral therapy. This group had a mean BCVA (LogMAR) of 0.875 ± 0.685 at the time of sample collecting and showed a significant improvement (*P*-value = 0.008) at last clinic visit with a mean BCVA (LogMAR) of 0.726 ± 0.793.

All corneal surgeries performed on the recruited patients were recorded. 75 patients (39%) underwent at least one corneal surgery. Amniotic membrane transplantation (AMT) was performed at least once in 45 eyes (23.2%). To determine a possible correlation between the comorbidities and a poor outcome, we divided the patients into 2 groups based on the BCVA (logMAR) at the last clinic visit: Group A with a BCVA > 0.4 and Group B with a BCVA ≤ 0.4. The second parameter for poor outcome was the need for corneal surgery. For this purpose, the patients were also divided into 2 groups: Group A with at least one corneal procedure and Group B without corneal surgery. The results for these tests are presented in Tables 11, 12, 13 and 14.

**Table 11:** Correlation between the ocular comorbidities and the final visual acuity

	BCVA > 0,4 (logMAR) (*n* = 95)	BCVA ≤ 0,4 (logMAR) (*n* = 90)	*P*-Value
Non-corneal eye surgery	45	13	< 0.001
Blepharitis	26	22	ns
Corneal surgery	28	8	< 0.001
Unspecified pervious eye infection	13	19	ns
Glaucoma	18	2	< 0.001
Wear of contact lenses	7	13	ns
Ocular Trauma	7	6	ns
Dry eye disease	3	2	ns
Other Keratopathy	3	4	ns
Fuchs’ endothelial dystrophy	5	2	ns
Unspecified conjunctivitis	2	2	ns
Ocular Pemphigoid	3	0	ns
Graft-versus-host disease	1	2	ns
Limbal stem cell deficiency	3	0	ns
Sjögren’s syndrome	0	2	ns
Entropion	1	1	ns
Keratoconus	2	0	ns
Ectropion	1	0	ns
Thyroid Eye Disease	1	0	ns
Stenosis of the lacrimal duct	1	0	ns
At least 1 Comorbidity	76	67	ns
At least 2 Comorbidities	55	16	< 0.001

*BCVA* best corrected visual acuity, *logMAR* Logarithm of the Minimum Angle of Resolution, *ns* not significant

**Table 12:** Correlation between the systemic comorbidities and the final visual acuity

	BCVA > 0,4 (logMAR) (*n* = 95)	BCVA ≤ 0,4 (logMAR) (*n* = 90)	*P*-Value
Atopic disease	10	10	ns
Immunosuppression	11	6	ns
Diabetes mellitus	12	4	0.048
Rheumatic diseases	6	4	ns
Previous tumor or malignant disease	6	2	ns
Rosacea	3	3	ns
Psoriasis	3	2	ns
Stem cell or organ transplantation	1	2	ns
Previous extraocular Herpes Infection	1	2	ns
HLA-B27 positivity	1	2	ns
Pityriasis rubra	1	0	ns
Human immunodeficiency virus positivity	0	1	ns
Acne	1	0	ns
Methicillin resistant staphylococcus aureus	0	0	ns
Chronic inflammatory bowel disease	0	1	ns
Sarcoidosis	0	1	ns
At least 1 Comorbidity	43	31	ns
At least 2 Comorbidities	13	7	ns

*BCVA* best corrected visual acuity, *logMAR* Logarithm of the Minimum Angle of Resolution, *HLA* human leukocyte antigen, *ns* not significant

**Table 13:** Correlation between the ocular comorbidities and the need for corneal surgery

	At least one corneal surgery (*n* = 75)	No corneal surgery (*n* = 119)	*P*-Value
Non-corneal eye surgery	37	25	< 0.001
Blepharitis	16	35	ns
Corneal surgery	30	7	< 0.001
Unspecific pervious eye infection	9	24	ns
Glaucoma	15	6	0.001
Wear of contact lenses	6	13	ns
Ocular Trauma	3	10	ns
Dry eye disease	1	6	ns
Other Keratopathy	5	2	ns
Fuchs’ endothelial dystrophy	6	1	0.009
Unspecific conjunctivitis	0	4	ns
Ocular Pemphigoid	2	1	ns
Graft-versus-host disease	0	3	ns
Limbal stem cell deficiency	2	1	ns
Sjögren’s syndrome	1	1	ns
Entropion	0	2	ns
Keratoconus	1	1	ns
Ectropion	1	0	ns
Thyroid Eye Disease	2	0	ns
Stenosis of the lacrimal duct	0	1	ns
At least 1 Comorbidity	62	89	ns
At least 2 Comorbidities	43	32	< 0.001

*ns* not significant

**Table 14:** Correlation between the systemic comorbidities and the need for corneal surgery

	At least one corneal surgery (*n* = 75)	No corneal surgery (*n* = 119)	*P*-Value
Atopic disease	10	11	ns
Immunosuppression	9	9	ns
Diabetes mellitus	9	10	ns
Rheumatic diseases	4	7	ns
Previous tumour or malignant disease	6	3	ns
Rosacea	1	6	ns
Psoriasis	3	2	ns
Stem cell or organ transplantation	0	3	ns
Previous extraocular Herpes Infection	0	3	ns
HLA-B27 positivity	0	3	ns
Pityriasis rubra	1	0	ns
Human immunodeficiency virus positivity	0	1	ns
Acne	0	1	ns
Methicillin resistant staphylococcus aureus	0	1	ns
Chronic inflammatory bowel disease	0	1	ns
Sarcoidosis	0	1	ns
At least 1 Comorbidity	33	46	ns
At least 2 Comorbidities	9	13	ns

*HLA* human leukocyte antigen, *ns* not significant

## Discussion

In our study, HSV-1 DNA was detected using conventional qualitative PCR in 18.6% of all eyes suspected of having HSK. A similar result was achieved by Lee et al., as they were able to detect HSV DNA in 20% of all tear samples taken in their study [18]. Guda et al. compared the sensitivity of different PCR methods [19]. They analyzed the corneal abrades from 50 eyes suspected of having HSK using both qualitative PCR and multiplex real-time PCR for HSV DNA and found a statistically significant difference in the sensitivity of both methods. In 78% of all suspicious eyes, the viral DNA was detected with multiplex real-time PCR, whereas a positive result was found in 40% of the suspicious eyes using qualitative PCR [19]. This shows the large difference in sensitivity of the various PCR methods. It also partially explains the difference between clinical suspicion rate and the rate of positive HSK-PCR results. It is also important to mention that the rate of PCR-positive findings is dependent on the type of specimen collected. The main specimen type in our study was corneal smears which contain hardly any corneal tissue, making the sensitivity of the PCR testing low compared to the PCR-sensitivity to HSK using corneal scrapings or corneal buttons after keratoplasty. Tóth et al. detected HSV-DNA in every 66 explanted cornea that showed no clinical suspicion of HSK referring to the high PCR-sensitivity to HSK when corneal buttons are tested [20]. Heo et al. reported a higher detection of HSV DNA in corneal scraping compared to tear specimen [21].

6 eyes (16.7%) in which the viral DNA was detected had a coinfection with other pathogens (bacteria or fungi). Erdem and colleagues published similar results when they found a co-infection with other pathogens in 24% of all eyes with detected HSV DNA [22]. Furthermore, they were able to detect HSV DNA in 9% of all eyes diagnosed with bacterial keratitis or fungal keratitis that did not show typical HSV lesions [22]. This underlines that HSK can be considered as a differential diagnosis in any keratitis, even if no typical HSK lesions are seen.

The most common ocular comorbidity in our patient population was previous non-corneal eye surgery, accounting for 32% of all eyes. Cho and colleagues reported about 15 eyes that developed epithelial keratitis after cataract surgery [23]. Chen et al. described PEDs after pars plana vitrectomy (ppV) and diagnosed four patients with HSK after retinal surgery [24]. Rao et al. reported four eyes that developed HSK or herpes keratouveitis after glaucoma surgery with application of mitomycin C (MMC), suggesting the virus-activating effect of MMC [25]. Blepharitis is an ocular surface disease and was the second most common ocular comorbidity, accounting for 26% of the eyes included in the study. Khoo and colleagues found an ocular surface disease in 18% of eyes with microbial keratitis [26]. Another common ocular comorbidity is previous corneal surgery, which was documented in 19% of the eyes in our group. Several studies confirmed this finding and described the new occurrence of HSK after pKPL. This can be explained by postoperative immunosuppression to reduce the risk of rejection but also due to the traumatic damage of the trigeminal nerve during surgery [27, 28]. It is also important to know that the reactivation of HSV after keratoplasty can imitate an endothelial allograft rejection. Abu Dail et al. detected HSV-DNA in 9 of 108 eyes with endothelial allograft rejection. Seven of these nine eyes had no previous history of HSK. This shows that HKS should always be considered as a differential diagnosis in clinical endothelial allograft rejection, even in cases with no previous history of HSK [29]. Crosslinking (CXL) is a possible trigger for HSV reactivation [30]. Several studies have described the occurrence of keratouveitis and microbial keratitis after this procedure. In addition to surgical trauma and the postoperative immunosuppressive effect of topical steroids, UV-A radiation, which is used in CXL, is a possible trigger for HSK reactivation, even in patients without a known ocular herpes infection [30–32]. Refractive surgery has also been noted in the literature as a possible trigger of HSK. The laser pulses used in phototherapeutic keratectomy (PTK) and microkeratome-assisted laser in situ keratomileusis (LASIK) are traumatic events that may reactivate a latent herpes infection [33, 34]. Approximately 11% of our patients had glaucoma at the time of sample collection. Different pathomechanisms contribute to the fact that glaucoma patients might be at risk for HSK: The corneal barrier function is compromised in glaucoma patients due to the ongoing inflammatory processes with the secretion of proinflammatory cytokines, which disrupts the corneal epithelial integrity by affecting the tight junctions between the corneal epithelial cells [35, 36]. On the other hand, subclinical inflammatory processes including the presence of proinflammatory cytokines in glaucoma patients might facilitate viral reactivation. Thomas et al. reported that proinflammatory cytokines play an important role in the manifestation and progression of herpes stromal keratitis [37]. Meibomian gland dysfunction has been reported to be associated with the long-term use of antiglaucomatous therapy, leading to a higher prevalence of ocular surface disease (OSD) with blepharitis and dry eye disease [38]. Several studies described a high prevalence of OSD in patients receiving antiglaucomatous eye drops [39–41]. Ghosh and colleagues reported the number of antiglaucomatous eyedrops and the duration of therapy as risk factors for clinical signs of OSD [39]. It has been shown that antiglaucomatous drops preserved with benzalkoniumchlorid (BAC) slow down corneal wound healing [42]. There is a discrepancy in the literature regarding a possible relation between antiglaucomatous therapy and the development or reactivation of HSK. Several case reports confirmed the new occurrence or reactivation of HSK after topical application of prostaglandin analogues [43, 44]. Other studies compared the effect of different antiglaucomatous drops on triggering HSK and found no correlation between prostaglandin analogues and HSK onset or reactivation compared to other antiglaucomatous agents [45, 46]. Contact lens (CL) wear was a common risk factor in our study, as 9.8% of all subjects had this comorbidity. This factor is mainly associated with microbial keratitis and was described as the most common comorbidity of microbial keratitis in the study by Green et al. with 22% of affected eyes [47]. Mucci et al. found that the recurrence rate of HSK is almost twice as high in CL wearers compared to non-CL wearers [48]. Three of our patients had chronic graft versus host disease (GvHD), which is associated with immune deviation, immune dysregulation and immunosuppression and often treated with immunosuppressants. Ocular chronic GVHD is a severe ocular surface disease with ongoing inflammatory activity in many cases. These factors as well as the topical therapy with steroids and/or cyclosporin contribute to the risk of HSV reactivation. We found active HSK in 6% of patients in a cohort of patients with ocular chronic GvHD, which indicates that HSK should always be considered especially in these patients [49].

Atopic diseases were the most common systemic comorbidity in our study, accounting for 10.8% of the included eyes. Borkar et al. reported that patients with atopy were 2.6 times more likely to develop ocular HSV disease (this included blepharitis, conjunctivitis, keratitis or uveitis) compared to non-atopic patients [50]. Marolo et al. reported an increase in ocular surface disease including corneal involvement in patients with atopic dermatitis treated with dupilumab [51]. Diabetes mellitus was one of the most frequently documented comorbidities in our study, affecting approximately 9% of the included eyes. Wang et al. compared corneal wound healing after cataract surgery in diabetic vs. non-diabetic patients. They reported impaired tear film quality, reduced corneal sensitivity and prolonged wound healing time in diabetic patients [52]. Kaisermann et al. documented a higher incidence of herpetic ocular surface disease in diabetic compared to non-diabetic patients [53]. In the study by Coco et al., diabetes mellitus was reported as a risk factor for PEDs in 20% of patients [54]. Approximately 10.2% of the eyes in our study had systemic immunosuppression as the second most common comorbidity in our population. In the study by Ting et al., systemic immunosuppression was documented as the third most common risk factor for the development of bacterial keratitis [55]. Schaftenaar et al. observed a high prevalence of HSK in HIV patients, partly as complicated forms as a bacterial co-infection or also as concomitant uveitis [56]. Rheumatic diseases were documented in 5.7% of the patients in our study. Yoshida et al. reported four rheumatoid arthritis patients who developed concomitant HSK with autoimmune-associated ulcerative keratitis and had a poor outcome [57].

Corneal erosion was the most common clinical manifestation in our study, presenting in 67.5% of all eyes examined. This is a common finding in both viral and microbial keratitis. Cabrera-Aguas et al. found corneal erosion in 85% of all eyes with mixed microbial and herpetic keratitis [58]. Corneal vascularization is a vision-threatening manifestation of HSK. This was documented in 36.6% of eyes in our study. In the study by Grubešić et al., this complication was recorded in 23.6% of all HSK eyes [59]. In our cohort, 10% of all eyes had a corneal scar at the time of sample collection. This percentage increased during follow-up and reached 33% of all eyes of patients who presented for follow-up 36 months after sample collection. Miserocchi et al. published a similar observation [60]. They recorded the complications of ocular HSV disease and found that approximately 12% of the eyes examined developed a corneal scar [60]. PEDs were observed in 14% of the eyes at the time of specimen collection. This percentage was unchanged 6 months after sampling and only decreased to approximately 7% and 2% at 12 and 24 months, respectively, indicating the severe, refractory course of this complication. In the study by Kim et al., about 17% of the eyes examined had persistent corneal erosion [61]. Another complication is ulcer formation, which was noted in 20% of the eyes at the time of sample collection in our study. With adequate antiviral therapy, this percentage decreased over time, as only 1% of eyes had an ulcer 6 months after sampling and no corneal ulcer was documented from the twelfth month after the samples were collected. According to our results, Das et al. reported a corneal ulcer or epithelial defect in 28.47% of the included eyes [62].

Although the viral DNA was only detected in approximately 18.6% of the eyes, topical and systemic antiviral therapy was used in around 43% and 41% of these eyes, respectively. Acyclovir tablets were the most common oral antiviral therapy. A preventive effect of oral acyclovir administration on the recurrence rate of ocular herpes disease has been reported in the literature [63]. Another important therapeutic aspect in the treatment of herpes-associated stromal keratitis is the use of topical steroids. In our study, approximately 46% of eyes were treated with topical steroids. Collum et al. confirmed the importance of topical steroids in the treatment of endothelial herpetic keratitis. They recorded faster healing and a higher healing rate with a combination therapy of topical acyclovir ointment with steroid drops compared to topical antiviral therapy alone [64]. However, topical steroid therapy should be initiated carefully, since it is contraindicated at first presentation with epithelial HSK.

Three months after sample collection, BCVA improved on average in our cohort. This tendency was not confirmed in the further follow-up, as the average visual acuity at the remaining follow-up examinations was not better than at the time of sample collection. This discrepancy may be due to the fact that the patients with more severe forms and thus worse visual acuity attended the follow-up appointments more frequently and accordingly lowered the average BCVA in the follow-up examinations. Shah et al. recorded the BCVA in HSK patients 6 weeks after the first visit and observed an improvement tendency [65]. Shrestha et al. recorded an improvement in BCVA one month after the first visit in patients with stromal HSK [66]. However, these observations need to be pursued in the long term to obtain definite conclusions. In our study, HSK recurrence was defined as new occurrence of infiltrates, epithelial bullae, corneal edema, endothelial precipitate or corneal vascularization. The HSK recurrence rate varied between 4.4% and 11.6% of the examined eyes. Miserocchi et al. recorded a recurrence as keratitis or keratouveitis in approximately 50% of all eyes with ocular HSV disease [60]. Kim et al. documented a recurrence rate of 32% of all eyes with HSK. They concluded, in fact, that the longer the follow-up, the higher the recurrence rate [61]. 75 eyes (39%) in our study required at least one corneal surgery. AMT was performed in 45 eyes (23.2%) and was the most common corneal procedure. Elhardt et al. compared the efficacy of AMT with that of pKPL in the treatment of corneal perforation. They found no statistically significant difference in perforation recurrence rate or final visual acuity [67]. This suggests that AMT may be a good alternative to pKPL in the treatment of small corneal perforations [67]; however in advanced ulcerations and extensive thinning and perforation, corneal grafting is often necessary. Dekaris et al. reported the good efficacy of AMT in the treatment of PEDs after pKPL [68]. We reported a beneficial effect of AMT simultaneously performed with pKPL in cases of severe corneal melting regarding the wound healing process [69]. Elective or emergency pKPL (including mini-pKPL) was performed a total of 43 times in our study. Holbach et al. reported a survival rate of 68% eleven years after corneal transplantation in eyes with HSK. They found that graft survival depended on HSK activity at the time of corneal transplantation [70]. In our group, eyes with previous ocular surgery, glaucoma or previous corneal surgery had a poor outcome. According to our results, a poor outcome of microbial keratitis has been reported in the literature in patients who had undergone ocular surgery and in patients who received topical steroid therapy [71, 72]. To our knowledge, we describe for the first time the association between glaucoma and clinical outcome in eyes with keratitis and corneal surface defects. We also found that diabetes mellitus was associated with a poor outcome. Almulhim et al. also demonstrated a correlation between diabetes mellitus and poor clinical outcome in eyes with bacterial keratitis [73]. Rathi et al. described uncontrolled diabetes mellitus as a possible risk factor for poor outcome in patients with polymicrobial keratitis. Nevertheless, this was a retrospective study with a small group of patients, referral bias and variation in treatment protocols before presentation, which means that further randomized studies with a larger group of patients are necessary to confirm these results [74].

## Conclusion

Our data confirm that HSK should be treated based on the clinical findings regardless of the PCR result. Patients with suspected HSK who underwent ocular or corneal surgery and those with glaucoma and diabetes mellitus should be closely monitored due to the risk of a poor outcome. Although corneal surgery was necessary in almost 40% of the eyes during the follow-up due to a complicated course, a significant overall improvement in visual acuity was achieved compared to the initial findings.

## Data Availability

The datasets used and/or analysed during the current study are available from the corresponding author on reasonable request.
